# A Real-Time Ultraviolet Radiation Imaging System Using an Organic Photoconductive Image Sensor†

**DOI:** 10.3390/s18010314

**Published:** 2018-01-22

**Authors:** Toru Okino, Seiji Yamahira, Shota Yamada, Yutaka Hirose, Akihiro Odagawa, Yoshihisa Kato, Tsuyoshi Tanaka

**Affiliations:** Panasonic Corporation, 1 Kotari-yakemachi, Nagaokakyo City, Kyoto 617-8520, Japan; yamahira.seiji@jp.panasonic.com (S.Y.); yamada.shota@jp.panasonic.com (S.Y.); hirose.yutaka@jp.panasonic.com (Y.H.); odagawa.a@jp.panasonic.com (A.O.); kato.yoshihisa@jp.panasonic.com (Y.K.); tanaka.tsuyoshi@jp.panasonic.com (T.T.)

**Keywords:** organic photoconductive film, CMOS image sensor, ultraviolet, hydrogen flame, corona discharge

## Abstract

We have developed a real time ultraviolet (UV) imaging system that can visualize both invisible UV light and a visible (VIS) background scene in an outdoor environment. As a UV/VIS image sensor, an organic photoconductive film (OPF) imager is employed. The OPF has an intrinsically higher sensitivity in the UV wavelength region than those of conventional consumer Complementary Metal Oxide Semiconductor (CMOS) image sensors (CIS) or Charge Coupled Devices (CCD). As particular examples, imaging of hydrogen flame and of corona discharge is demonstrated. UV images overlapped on background scenes are simply made by on-board background subtraction. The system is capable of imaging weaker UV signals by four orders of magnitude than that of VIS background. It is applicable not only to future hydrogen supply stations but also to other UV/VIS monitor systems requiring UV sensitivity under strong visible radiation environment such as power supply substations.

## 1. Introduction

Imaging ultraviolet radiation during ignition and discharge of excited molecular species is of high value for safety monitoring systems of power plants such as hydrogen stations and transformer substations. For instance, although construction of hydrogen stations has already been started in several countries [1], a major concern lies in the fact that hydrogen becomes extremely flammable due to lowering the ignition energy when mixed with air in a relative contents range of 10% to 60% [2]. Furthermore, given the fact that the emission band head of the hydrogen flame lies in an ultra-violet (UV) region (~310 nm), it is invisible to human eyes. A worse and reported scenario is that human beings pass through the invisible hydrogen flame caused by accidentally leaking hydrogen from a high pressure tank ignited near the leaking point [1]. Also, when cables of power supply stations become degraded, leakage of high power radiation causes corona discharge of air resulting not only in human hazard but also in a fatal accident.

In these systems, it is important to immediately and accurately detect failing locations that emit weak (possibly 1–10 nW/cm^2^) UV lights superposed on ordinary background daylight (10^5^ lux). This means that the required imaging system should be able to visualize both UV and visible (VIS) light scenes preferably without using optical UV filters on account of system cost and size. In this regard, the sensitivity of ordinary Si CMOS image sensor (CIS) in UV region is not sufficiently high although there exist a specially made CIS or Si-based photodetectors in which the quantum efficiency of UV light is enhanced, they require additional cost for the special fabrication process [3,4,5]. In addition, although the UV sensing material, i.e., AlGaN, based sensors with high sensitivity in a UV range have been reported, they are incapable of imaging VIS light [6,7].

Recently, to resolve the above issues, we reported an optical filter-less ultraviolet and visible (UV + VIS) imaging system based on an organic photoconductive film (OPF) imager [8]. In this work, we report the system design issues and the performances of the above system in more details. The paper is organized as follows. Firstly, advantages of the OPF imager in terms of intrinsic sensitivity in the entire spectral region desired, i.e., from UV to VIS regions, and of pixel circuit configuration are given. Secondly, signal processing scheme of subtracting a high level background VIS image from a UV hydrogen flame image overlapped on the background is described. The operation is performed frame-by-frame and, therefore, in real time. Then, experimental results of hydrogen flame imaging superposed on a high level background is given. It is shown that the system is capable of imaging a weak hydrogen flame signals from four orders of magnitude higher VIS background signals. Also, imaging results of a corona discharge phenomenon is given. Finally, a brief conclusion is given.

## 2. Advantage of OPF Imager

**Figure 1:**
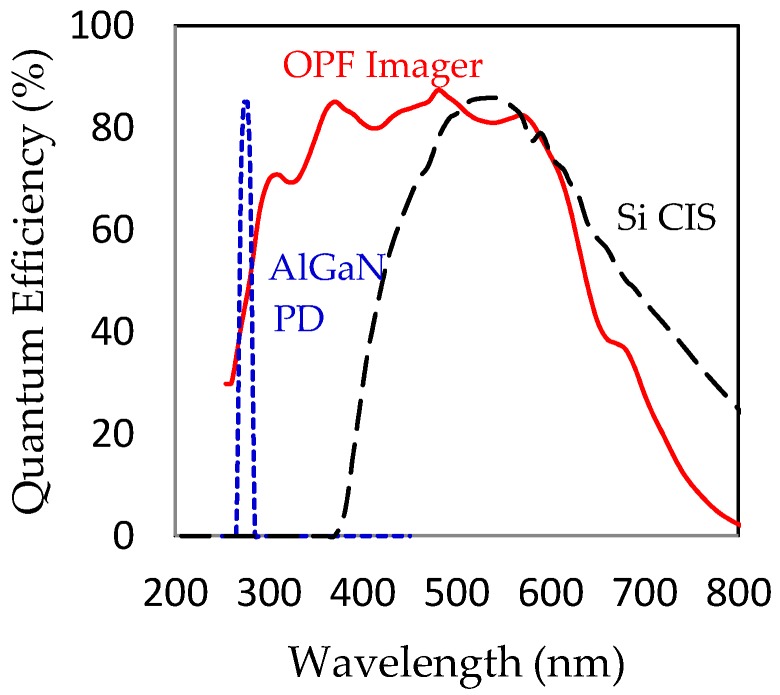
Quantum efficiency spectra of the present OPF CMOS imager (red), a Si CIS (black) and an AlGaN photodiode (blue) [7].

Major advantages of the OPF imager as the hydrogen imager are two folds. Firstly, as shown in Figure 1, the OPF imager has a high quantum efficiency both in the VIS and the UV regions as compared with those of ordinary CIS or reported UV sensing materials.

**Figure 2:**
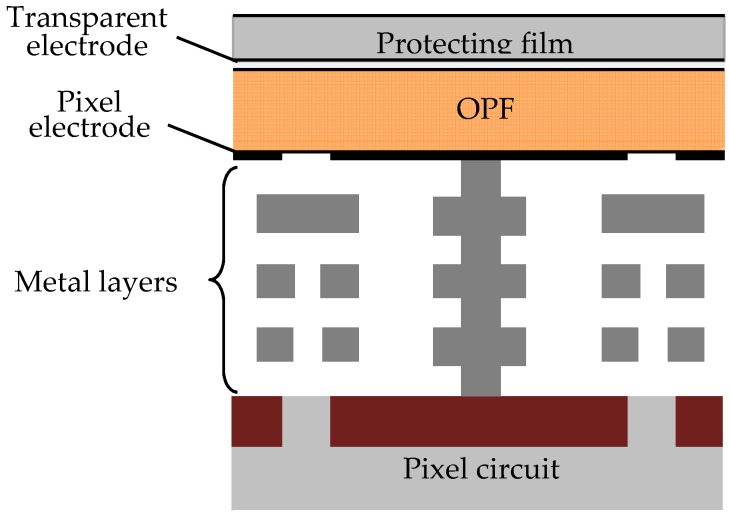
A cross sectional view of an organic photoconductive film (OPF) CMOS imager.

**Figure 3:**
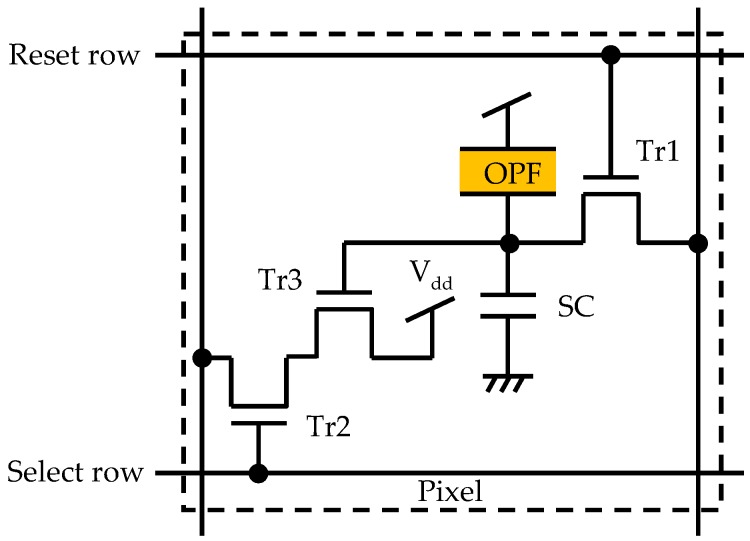
A schematic of the pixel circuit.

The schematic cross sectional view of a typical pixel region of the OPF imager is shown in Figure 2. The device consists of, from the top in order, a protecting film, the top transparent electrode, OPF, and the bottom pixel electrode directly connected to CMOS circuits [9,10]. Since OPF has a high absorption coefficient both for UV light and VIS light, the thickness of OPF could be reduced to 0.5 μm from that of the ordinary photodiodes depth of CIS, i.e., 2–3 μm, while still keeping a high sensitivity. Secondly, a high dynamic range of the OPF due to separation of the storage node from the photo-conversion area can be used to accommodate the high level VIS background. The pixel circuit is 3-Tr (a reset transistor (Tr1), a selection transistor (Tr2), and a source follower amplification transistor (Tr3)) configuration and in order to extend the saturation level to the highest level possible, a storage capacitance (SC) of large capacity is incorporated as shown in Figure 3. The SC allows a charge accumulation up to the breakdown voltage of the junction, the input of Tr3 can be swung to the level of the input voltage of Tr3 (V_dd_) giving rise to a much higher saturation level [10]. The specifications of the developed OPF imager are summarized in Table 1.

## 3. Proposed Imaging System

**Figure 4:**
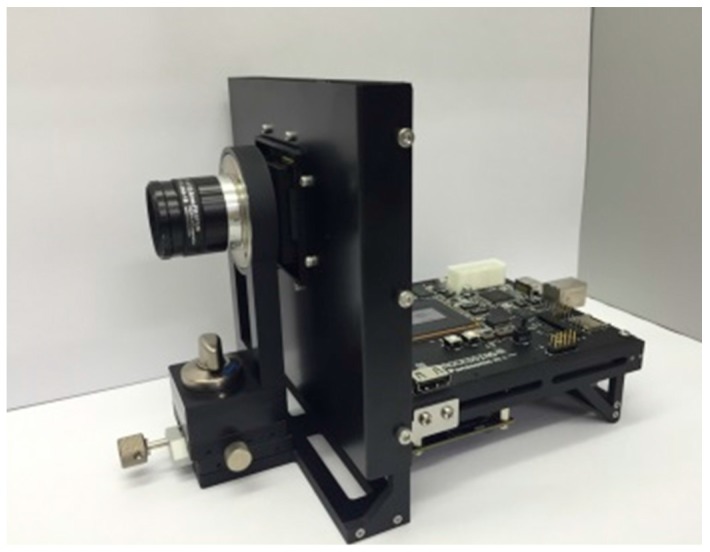
A photograph of the developed imaging system (camera).

**Figure 5:**
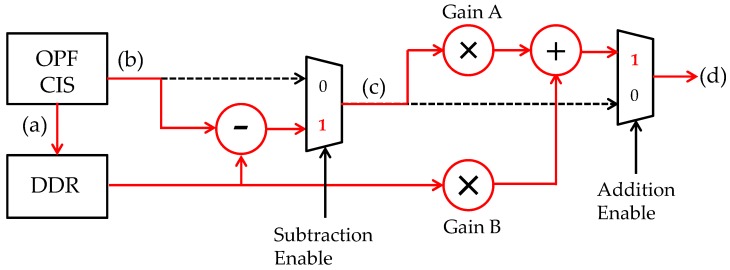
A signal flow diagram of the developed UV imaging system. (a) is the reference visible light background image. (b) is the UV image of interest overlapped on the VIS background image. (c) is the UV image extracted by subtracting (a) from (b). (d) is the synthesized image extracted by adding (a) to amplified (c).

A photograph of the developed imaging system (camera) is shown in Figure 4. It consists of three boards, i.e., a power supply board, a signal processing (field programmable gate array (FPGA)) board, a sensor head board on which a c-mount lens is attached. Figure 5 shows a signal flow diagram embedded in the FPGA. To explain the flow of signal processing in order, first, the reference visible light (VIS) image data are taken right before the UV + VIS images capture starts, and held in a double-data-rate (DDR) synchronous dynamic random access memory (SDRAM). Secondly, a UV image of interest overlapped on the VIS background image is captured. Thirdly, only the UV image is extracted by subtracting a VIS reference image stored in the DDR from the UV + VIS image. Then, the UV image signal is amplified. Finally, the reference visible image (VIS) is synthesized with the extracted UV image. With this method, it is possible to visualize the UV light without optical filter and to determine the UV emission points in real time and in a VIS environment (background). Although, strictly speaking, a background VIS environment illumination condition might change during the UV + VIS imaging operation, the effect is practically avoided by occasional “refreshing” reference images operation.

## 4. Results and Discussion

(1) Imaging Hydrogen Flames

**Figure 6:**
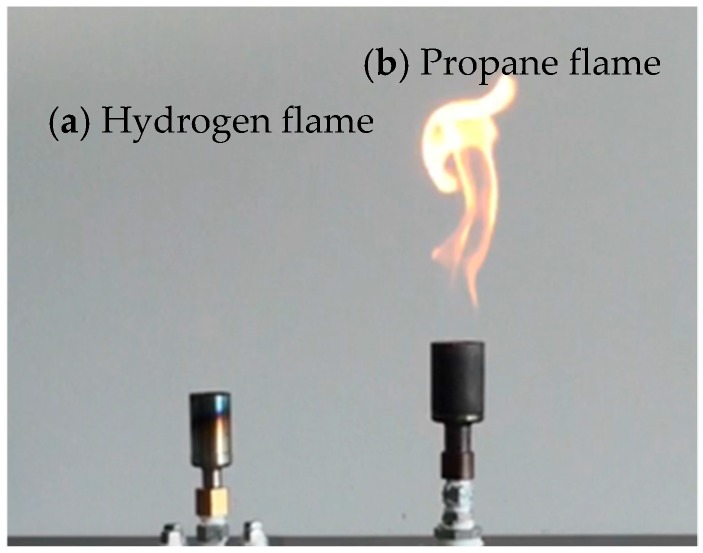
Flame images of hydrogen (**a**) and propane (**b**) taken by an ordinary CIS showing entirely invisible characteristics of the hydrogen flame.

**Figure 7:**
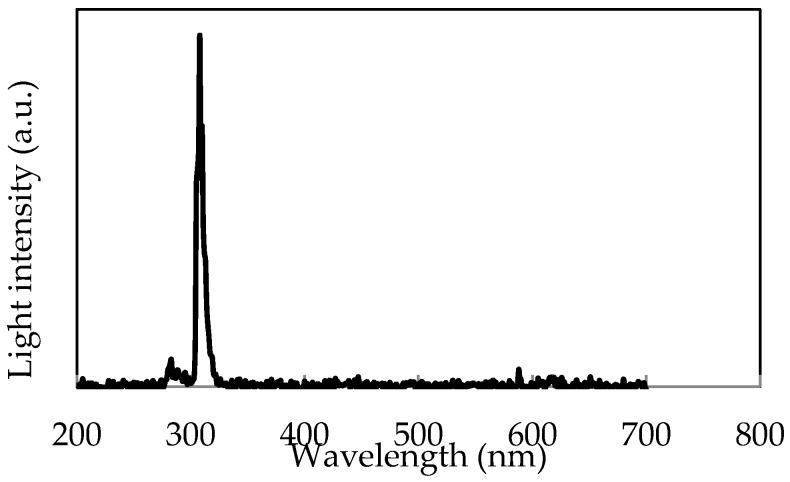
Light intensity spectrum of the hydrogen flame with a peak at around 310 nm.

In the first experiments, we demonstrate hydrogen flame visualization. As flame sources, Bunsen burner systems of hydrogen and of propane are used. The distance between the camera and hydrogen flame is 8 m. In Figure 6, flames are lit on both burners. The image is taken with a visible CIS. The propane flame (right side) with orange emission is clearly visible while the hydrogen flame (left side) is entirely invisible. A daylight irradiation at about 1000 lux of halogen lamp (color temperature: 2800 K) was measured with an illuminance meter (CL-500A, Konica Minolta, Tokyo, Japan). The spectrum of hydrogen flame was measured with a spectrometer (SPG-120UV, Shimadzu, Kyoto, Japan) is shown in Figure 7. Intensity of the UV radiation is ~10 nW/cm^2^ was calibrated by Si-PD (S12698, Hamamatsu Photonics, Shizuoka, Japan) and a high transmission UV bandpass filter, i.e., the pass band (FWHM) of 310 nm ± 5 nm and OD5.

**Figure 8:**
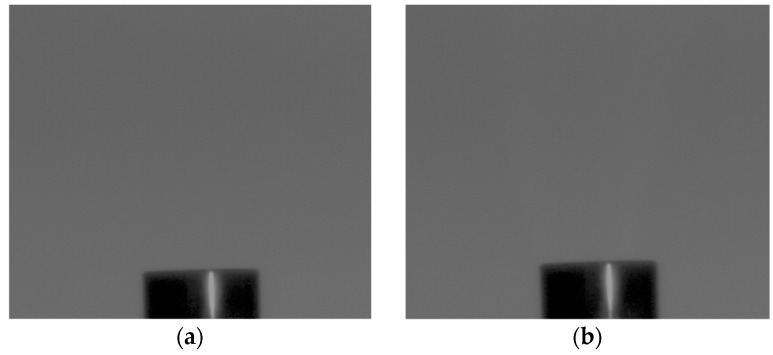

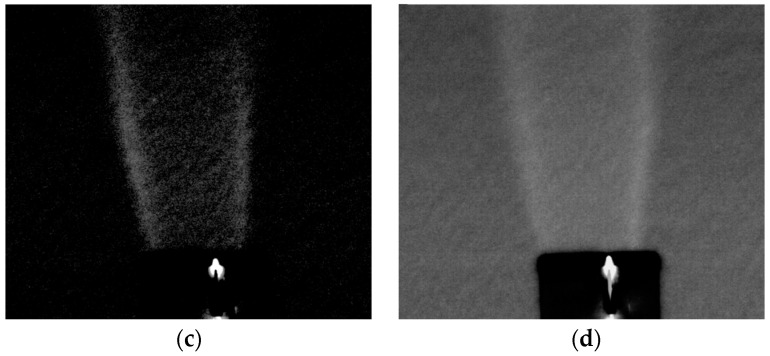
Images of tip of burner: (**a**) is a reference visible light (VIS) image stored in the DDR; (**b**) is an image including the UV light of hydrogen flame and background visible light (VIS + UV); (**c**) is the hydrogen flame image extracted by subtracting (**a**) VIS image from (**b**) UV + VIS image; (**d**) is the hydrogen flame image extracted by synthesizing (**a**,**c**).

In Figure 8, obtained images at the specified nodes of the signal flow diagram in Figure 5 are shown. Figure 8a is a reference visible light (VIS) image stored in the DDR. Figure 8b is an image including the UV light of hydrogen flame and background visible light (VIS + UV). Here, hydrogen flame cannot be recognized because the intensity of hydrogen flame is smaller by four orders of magnitude with respect to the background VIS light. We have extracted a hydrogen flame image Figure 8c by subtracting Figure 8a VIS image from Figure 8b, i.e., UV + VIS image. Finally, the hydrogen flame image of Figure 8c is synthesized with the reference image of Figure 8a resulting in the clear image of Figure 8d.

**Figure 9:**
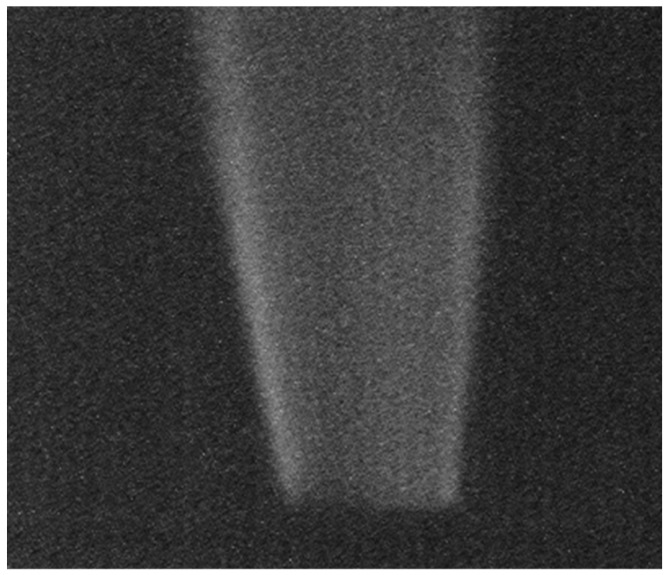
UV image of hydrogen gas extracted by an UV optical filter system.

The signal to noise ratio (SNR) of the image in Figure 8b is −32 dB due to dominance of the background visible light. By subtracting the VIS signal, SNR is recovered to +3 dB or 35 dB improvement. We also took a hydrogen flame image using a UV narrow band filter as a comparison. Although it is highly dependent on the performance of the filter, with use of the same filter by using the same filter as that used for calibration with the Si-PD, an image with 12 dB SNR is obtained as shown in Figure 9. We note that the filter-less system we developed is preferred because of cost benefit. However, it is also noted that a higher S/N image than +3 dB is desirable and will be realized by designing a larger pixel size imager in the future.

**Figure 10:**
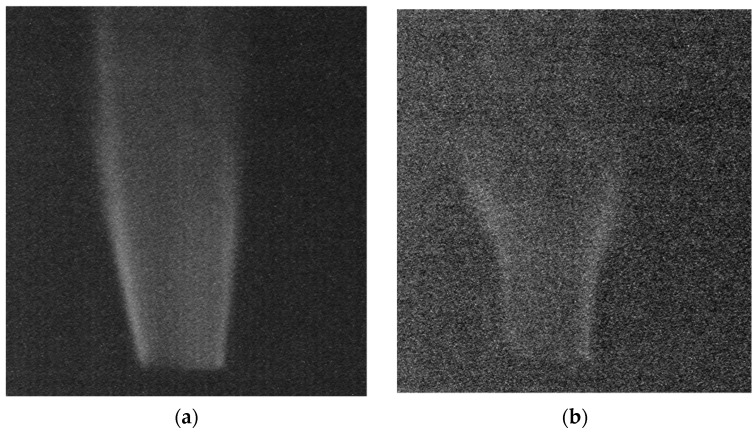
UV images of hydrogen gas generated by a rolling shutter mode (**a**) and by a global shutter mode (**b**).

Finally, in order to investigate a dynamic motion of the hydrogen flame, imaging by a global shutter (GS) operation mode [11] is tested as shown in Figure 10. The synchronous global shutter capture of the OPF CIS is preferred over an ordinary CIS operating in “quasi-” GS or high speed rolling shutter mode, since it avoids image distortion. Compared with an image taken with a rolling shutter mode (left), an image taken with a GS mode (right) exhibits a more resolved signature presumably due to dynamic motion of the flame such as turbulence. It is expected to play a useful role in the investigation of flame dynamics in future.

(2) Imaging Corona Discharge

**Figure 11:**
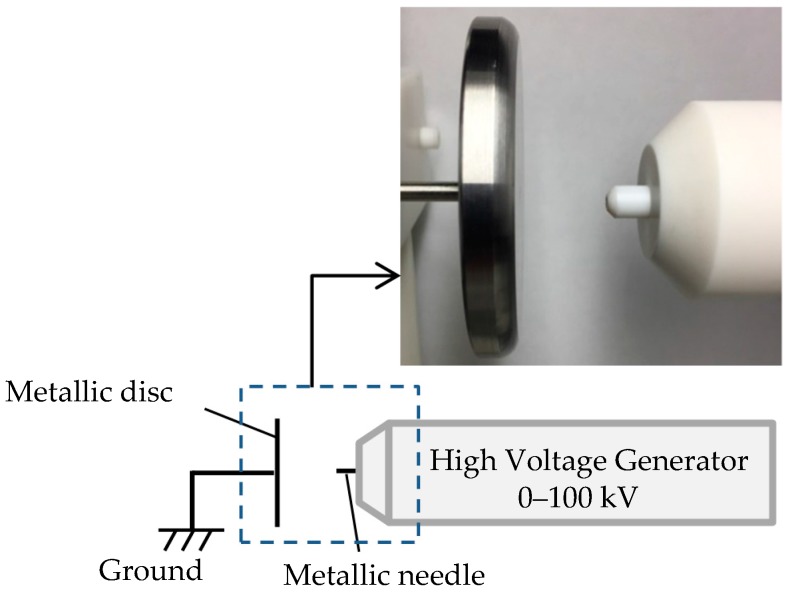
Diagram of the Experimental equipment for corona discharge observation.

**Figure 12:**
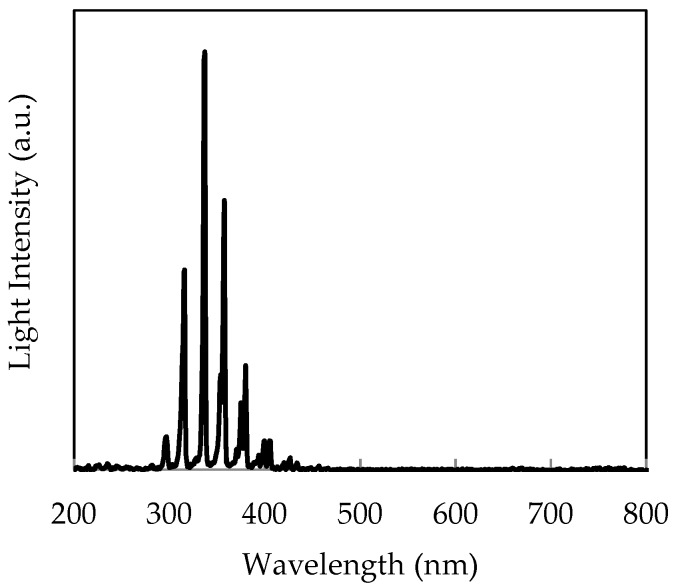
Light intensity spectrum of the corona discharge.

**Figure 13:**
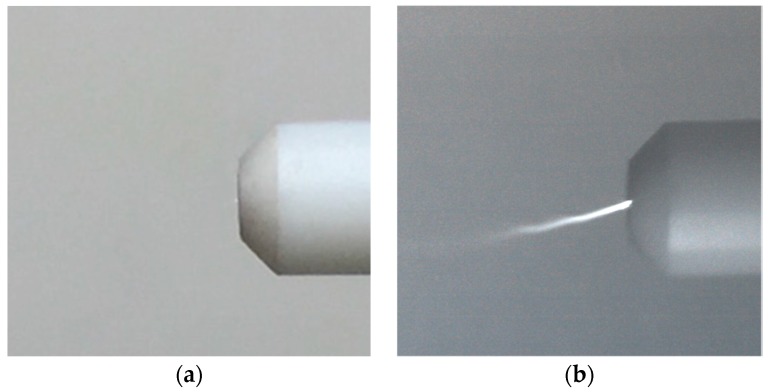
Images of corona discharge taken by an ordinary CIS (**a**) and an OPF imager (**b**).

As the second demonstration, we describe the usefulness of the system for discharge detection. A schematic diagram of the experimental setup for corona discharge observation is shown in Figure 11. It consists of a high voltage generator and metal electrodes, i.e., a flat metallic disc and a needle. The spectrum of corona discharge was measured with a spectrometer (PMA-12, Hamamatsu Photonics, Shizuoka, Japan), as shown in Figure 12. The spectral peaks due to the second positive transitions of nitrogen molecules (*C*^3^Π*u* → *B*^3^Π*g*) are clearly observed in the wavelength region from 300 nm to 400 nm [12]. The intensity of the UV radiation is 37 nW/cm^2^, whereas the background level is about 400 lux. As expected, an ordinary CIS, which is not equipped with the signal operation described in Figure 5, apparently failed to image the discharge radiation due to interference of the background as shown in Figure 13a. On the contrary, the discharge plasma radiation is clearly imaged by the present UV imaging system as shown in Figure 13b after subtraction of the background and later synthesis of the UV and the VIS images.

## 5. Conclusions

We have developed an imaging system using an OPF imager with high sensitivity and wide dynamic range capable of visualizing invisible hydrogen flame. We have employed a real-time monitoring system with a built-in filter function in the FPGA that extracts UV images by subtracting a VIS reference image from image including UV and VIS images. We demonstrated imaging of invisible hydrogen flames and a corona discharge plasma. This system is expected to be applicable to other UV imaging applications requiring UV sensitivity under a strong visible radiation environment.

## Figures and Tables

**Table 1:** Specification of the OPF imager used in the present work.

Process Technology	65 nm CMOS (1Poly-3Cu-1Al)
Number of pixels	1880 (H) × 1400 (V)
Pixel size	0.9 μm
Power supply	2.8 V, 10 V
Frame rate	10 fps
Saturation Charge	6500 electrons
Dynamic range	68 dB

## Abbreviations

The following abbreviations are used in this manuscript:

CMOS	Complementary Metal Oxide Semiconductor
UV	Ultraviolet
VIS	Visible
OPF	Organic photoconductive film
CIS	CMOS image sensor
CCD	Charge Coupled Device
Tr	Transistor
SC	Storage capacitance
FPGA	Field programmable gate array
DDR	Double-data-rate
SDRAM	Synchronous dynamic random access memory
SNR	Signal to noise ratio
FWHM	Full width at half maximum
OD	Optical density
GS	Global shutter
